# Caspofungin paradoxical growth in *Candida albicans* requires stress pathway activation and promotes unstable echinocandin resistance mediated by aneuploidy

**DOI:** 10.3389/fcimb.2025.1618815

**Published:** 2025-09-08

**Authors:** Ying Wei, Jing Wang, Nan Tang, Ziwei Lin, Wenhui Li, Yi Xu, Liangsheng Guo

**Affiliations:** ^1^ Department of Obstetrics and Gynecology, The Second Affiliated Hospital of Soochow University, Suzhou, China; ^2^ Department of Pharmacy, Zibo Zhoucun People’s Hospital, Zibo, China; ^3^ Department of Public Health, Zibo Zhoucun People’s Hospital, Zibo, China; ^4^ Department of Pharmacy, The 960th Hospital of PLA, Jinan, China

**Keywords:** *Candida albicans*, paradoxical growth, stress pathway, aneuploidy, transient resistance

## Abstract

Paradoxical growth (PG) is a counterintuitive phenomenon in which otherwise susceptible fungal cells resume proliferation at supra-MIC concentrations of echinocandins, thereby undermining the efficacy of these frontline antifungals. Despite its clinical significance, the genetic basis of PG remains poorly understood. Here, we systematically dissect the roles of key stress response pathways—Hsp90, PKC, calcineurin, and TOR—in mediating caspofungin (CSP)-induced PG in *Candida albicans* and uncover a novel genetic mechanism involving segmental aneuploidy. Disruption of these pathways via pharmacological inhibition or targeted gene deletion abolished PG, confirming their essential roles in mediating adaptive stress responses. Whole-genome sequencing of CSP-tolerant isolates revealed a recurrent segmental monosomy on the right arm of Chromosome R (SegChrRx1). Phenotypic reversion analyses demonstrated that CSP resistance is reversible and directly linked to the presence of this aneuploidy. Transcriptomic profiling of SegChrRx1 strains showed broad transcriptional remodeling, including upregulation of *GSC1* (encoding β-1,3-glucan synthase), *CHS3* and *CHS4* (chitin synthases), and key regulators of the PKC and calcineurin pathways, alongside downregulation of dosage-sensitive genes whose deletion enhances CSP resistance. Collectively, our findings reveal a dual mechanism of PG: activation of stress response pathways confers initial survival, while segmental aneuploidy enables reversible transcriptional reprogramming that promotes drug resistance. This study establishes segmental aneuploidy as a dynamic and previously underappreciated mechanism of echinocandin adaptation in *C. albicans*, with important implications for antifungal therapy.

## Introduction

Invasive fungal infections caused by *Candida albicans* remain a major source of morbidity and mortality, particularly in immunocompromised individuals (Pfaller and Diekema, 2007). Echinocandins—such as caspofungin (CSP), micafungin (MCF), and anidulafungin (ANF)—have emerged as first-line antifungal agents due to their fungicidal activity and selective inhibition of β-1,3-glucan synthase, a critical enzyme in fungal cell wall biosynthesis (Pappas et al., 2016). However, the clinical efficacy of echinocandins is increasingly compromised by the emergence of resistance mechanisms (Kanafani and Perfect, 2008). While paradoxical growth (PG) has been well-documented *in vitro*, its clinical relevance remains to be fully elucidated [reviewed in (Wagener and Loiko, 2017)].

PG refers to regrowth of *Candida* spp. at higher but not lower supra-MIC concentrations of echinocandins (Valero et al., 2020). This phenomenon has been consistently observed in *C. albicans* and other fungal species (Toth et al., 2020). PG has been linked to cell wall remodeling, particularly increased chitin deposition (Walker et al., 2013), and activation of conserved stress response pathways, including the PKC, calcineurin, and TOR signaling cascades. Heat shock protein 90 (Hsp90) plays a central role in stabilizing these pathways and has been shown to be essential for PG in both *C. albicans* and *Aspergillus fumigatus* (Reinoso-Martin et al., 2003; Lesage et al., 2004; Fortwendel et al., 2009; Singh et al., 2009; Lamoth et al., 2014; Caplan et al., 2018; Rivera and Heitman, 2023; Lefranc et al., 2024).

Beyond transcriptional responses, *C. albicans* exhibits remarkable genomic plasticity, often employing chromosomal aneuploidy to rapidly adapt to antifungal stress (Selmecki et al., 2006; Yang et al., 2013, Yang et al., 2017, Yang et al., 2019; Li et al., 2022; Sun et al., 2023; Yang et al., 2023; Zheng et al., 2023; Guo et al., 2024; Zheng et al., 2024). Whole-chromosome aneuploidies have been implicated in resistance to azoles and other stressors, yet the role of segmental chromosomal changes in mediating resistance remains largely unexplored. Segmental aneuploidy offers a mechanism for fine-tuned adaptation with potentially lower fitness costs compared to whole-chromosome alterations. The transient and reversible nature of PG suggests the involvement of epigenetic or unstable genetic mechanisms. Given that aneuploidy can reversibly alter gene dosage and broadly rewire cellular pathways, we hypothesized that segmental aneuploidy may contribute to PG in *C. albicans*.

In this study, we combined genetic perturbation, pharmacological inhibition, whole-genome sequencing, and transcriptomic profiling to dissect the molecular basis of CSP-induced PG. We demonstrate that PG arises from a dual mechanism: (1) activation of conserved stress response pathways that mediate immediate resistance, and (2) acquisition of a recurrent segmental aneuploidy on Chromosome R that promotes genome-wide transcriptional remodeling. These findings uncover a novel paradigm for antifungal resistance and underscore the therapeutic potential of targeting aneuploidy-stabilizing mechanisms or stress pathway hubs.

## Materials and methods

### Strains and growth conditions

The *C. albicans* reference strain SC5314 was used as the progenitor for this study. Gene knock strains are listed in **Supplementary Table S1**. Stock cultures were preserved in 25% glycerol and stored at -80°C. Cells were routinely cultured in Yeast Extract-Peptone-Dextrose (YPD) medium, which contains 1% (w/v) yeast extract, 2% (w/v) peptone, and 2% (w/v) D-glucose, at 37°C using a shaking incubator set to 150–200 rpm. Caspofungin (HY-17006), anidulafungin (HY-13553), micafungin (HY-16321), rapamycin (HY-10219), and NVP-HSP990 (HY-15190) were purchased from MedChemExpress (MCE). Drug solutions were prepared in dimethyl sulfoxide (DMSO) and stored at -20°C.

### Broth microdilution assay

Broth microdilution assay was performed according to the CLSI guidelines (CLSI, 2017) with minor modifications. Briefly, fungal cells were suspended in YPD broth and adjusted to a density of 2.5 × 10³ cells/mL. A serial dilution of echinocandins was tested across a concentration range of 0.0125–12.8 μg/mL. The inoculated 96-well plates were incubated at 37°C for 48 hours. Paradoxical growth was assessed by measuring optical density at 600 nm (OD_600_) using a NanoPhotometer (Implen, California, USA). PG was defined as fungal growth in consecutive wells at supra-MIC concentrations after the incubation period (Chamilos et al., 2007).

### Spot assay

**Figure 1:**
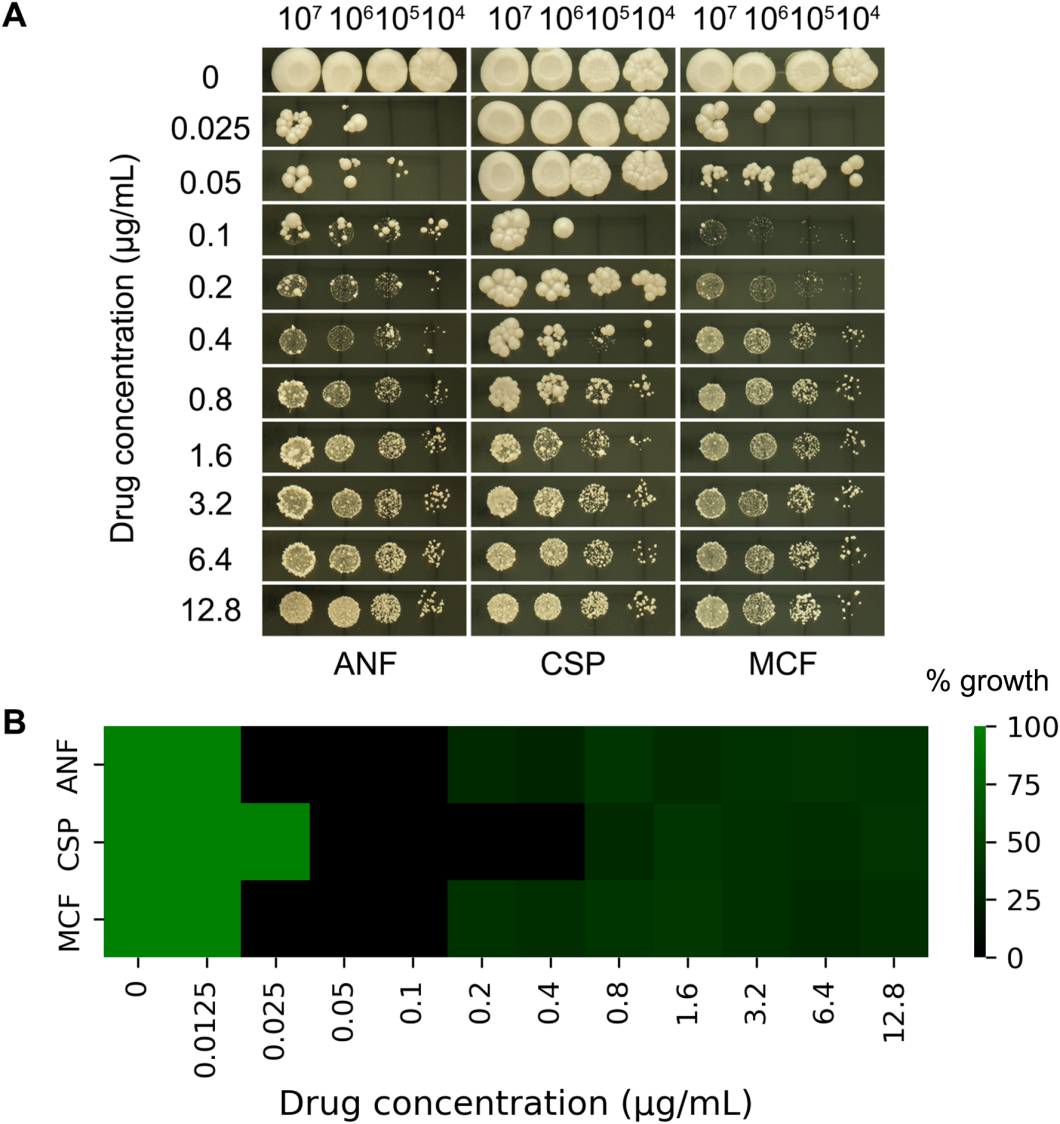
Paradoxical growth of **(*C*)** *albicans* in response to echinocandins. **(A)** Spot assay: Serial 10-fold dilutions (3 µL) of cultures were spotted onto YPD agar plates containing anidulafungin (ANF), caspofungin (CSP), or micafungin (MCF) at concentrations ranging from 0.025 to 12.8 µg/mL. Plates were incubated at 37°C for 48 hours. **(B)** Broth microdilution assay: Cells were grown in YPD broth in 96-well plates containing ANF, CSP, or MCF at concentrations ranging from 0.0125 to 12.8 µg/mL. Plates were incubated at 37°C for 48 hours, and growth was quantified by measuring OD_600_. Values were normalized against the drug-free control well.

**Figure 2:**
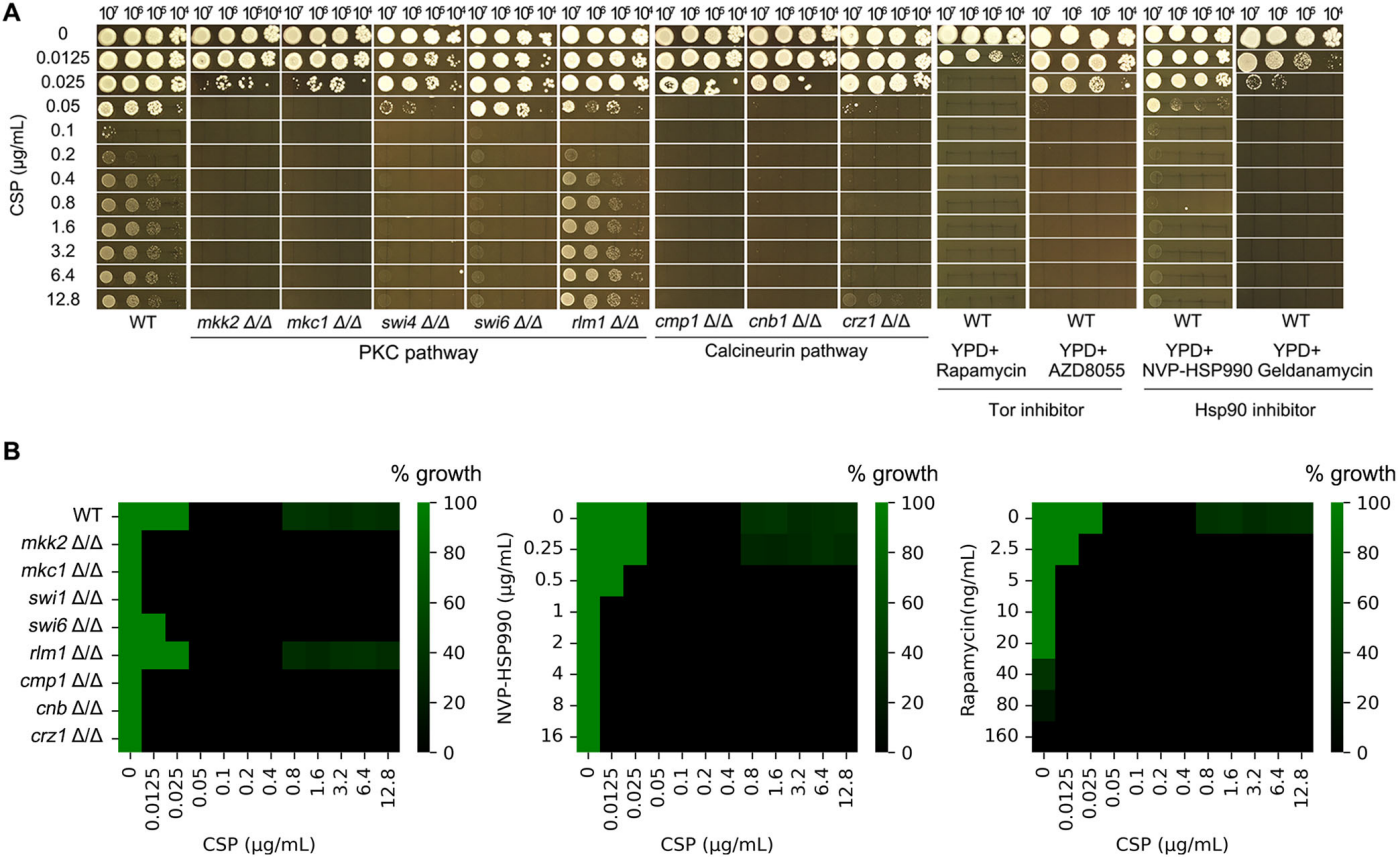
Genetic and pharmacological requirements for caspofungin paradoxical growth in (*C*) *albicans*. **(A)** The wild-type (WT) strain SC5314 was tested on YPD plates containing caspofungin (CSP; 0.0125-12.8 μg/mL) with or without inhibitors of key signaling pathways: TOR inhibitors rapamycin (10 ng/mL) and AZD8055 (4 μg/mL) or Hsp90 inhibitor NVP-HSP990 (1 μg/mL) and geldanamycin (0.5 μg/mL). Additionally, deletion strains of the PKC and calcineurin pathways were examined under the same conditions. All plates were incubated at 37°C for 48 hours before imaging. **(B)** Requirement of stress pathways for maintenance of PG evaluated with liquid assay. (Left) Broth microdilution assay of PKC pathway mutants (*mkk2Δ/Δ*, *mkc1Δ/Δ*, *swi4Δ/Δ*, *swi6Δ/Δ*, r*lm1Δ/Δ*), and alcineurin pathway mutants (*cmp1Δ/Δ*, *cnb1Δ/Δ*, *crz1Δ/Δ* CSP (0.0125-12.8 μg/mL). (Middle) WT (SC5314) exposed to CSP (0.0125-12.8 μg/mL) with HSP90 inhibitor NVP-HSP990 at concentrations indicated. (Right) WT (SC5314) exposed to CSP (0.0125-12.8 μg/mL) with Tor inhibitor rapamycin at concentrations indicated. Growth after 48 h at 37°C was quantified by OD_600_ and normalized to drug-free controls.

**Figure 3:**
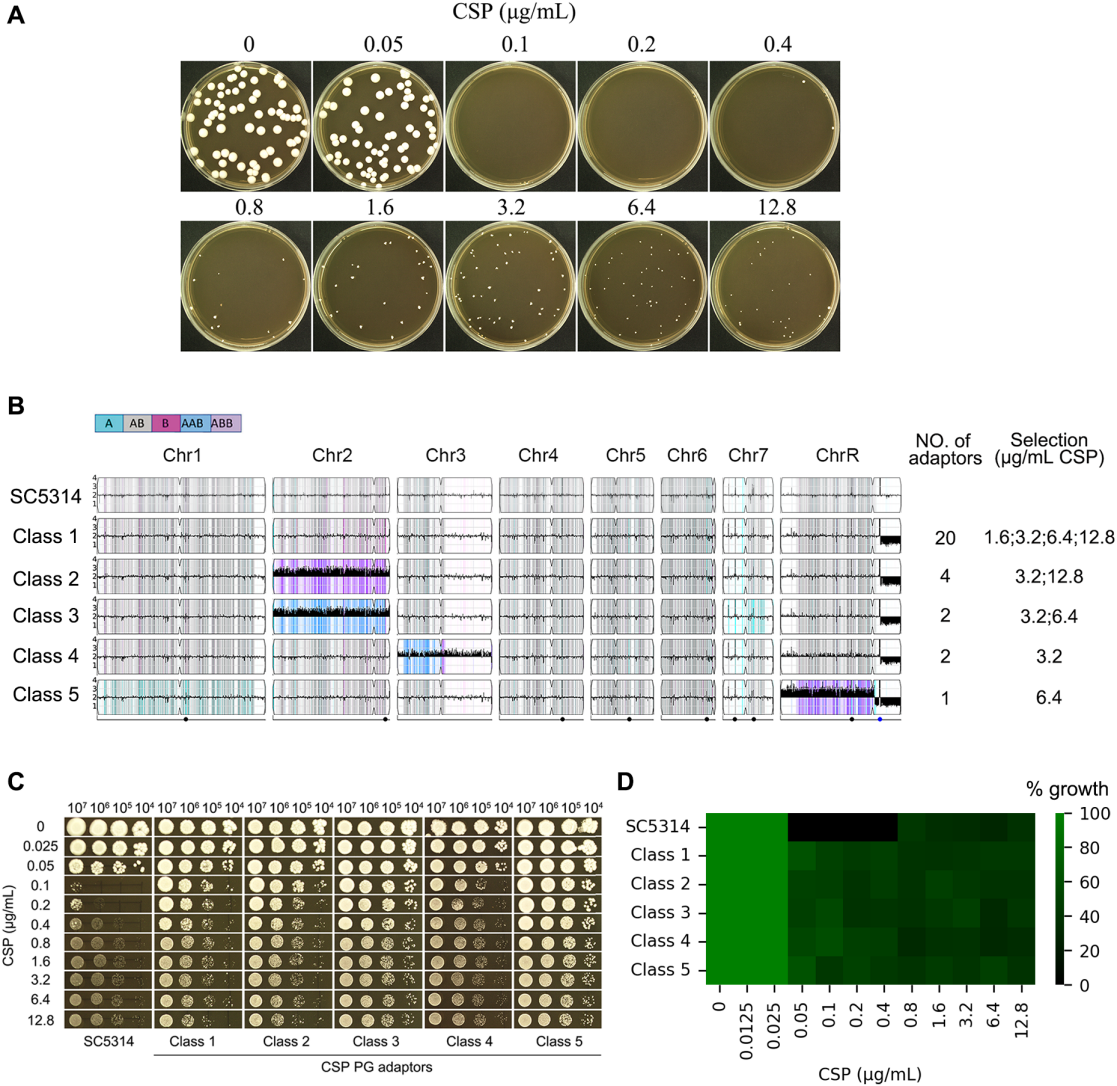
Paradoxical growth promotes caspofungin resistance through aneuploidy formation. **(A)** Isolation of adaptor strains: Approximately 50 SC5314 cells were plated on YPD agar containing caspofungin (CSP; 0.05-12.8 μg/mL) and incubated at 37°C for 5 days. All resulting colonies were collected for analysis. **(B)** Genomic characterization: 29 CSP-tolerant adaptor strains were sequenced. Karyotype analysis revealed these adaptor strains clustered into 5 distinct classes. The figure displays the distribution of adaptors across classes and their selection conditions. **(C, D)** Phenotypic validation: Representative adaptor strains from each class were assessed for CSP resistance using spot assays **(C)** and broth microdilution assay **(D)**.

**Figure 4:**
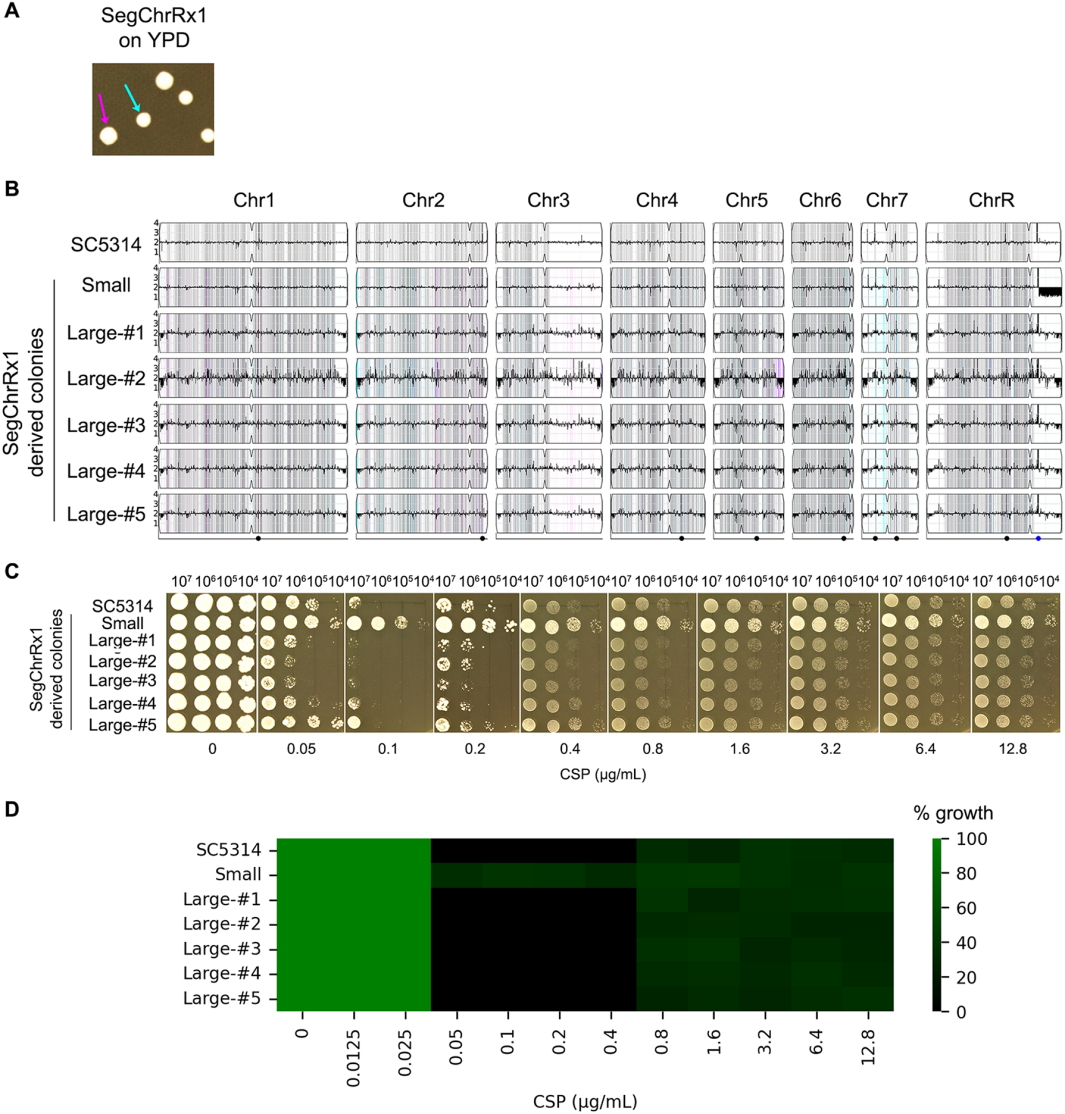
Phenotypic and genomic instability of aneuploid strains. **(A)** Colony morphology variation. A SegChrRx1 adaptor strain grown on YPD agar for 36 hours exhibited colony size polymorphism, with distinct small (cyan arrow) and large (magenta arrow) colonies. **(B)** Karyotype analysis. Whole-genome sequencing revealed that the progenitor SC5314 and all large colonies maintained euploid diploid genomes, while the small colony retained the SegChrRx1 aneuploidy. **(C, D)** Drug sensitivity profiling. Spot assays on YPD containing 0.05-12.8 μg/mL caspofungin **(C)** and broth microdilution assay using 0.0125-12.8 μg/mL CSP **(D)** showed complete inhibition of SC5314 and large colonies at lower supra-MIC CSP concentrations but PG at higher supra-MIC concentrations, while the small SegChrRx1 colony displayed ability to grow at both lower and higher supra-MIC CSP concentrations.

Cells were suspended in distilled water and adjusted to a concentration of 1 × 10^7^ cells/mL. 3 μL of 10-fold serial dilutions were spotted on YPD plates containing the indicated drug concentrations, with specific compounds and doses shown in **Figures 1**–**3** top and bottom panels, and **Figure 4** top right panel. The plates were incubated at 37°C and photographed after 48 hours.

### Obtaining PG adaptor strains

SC5314 cells were suspended in distilled water, and cell density was determined using a hemocytometer. Approximately 50 cells were plated on YPD agar supplemented with CSP (0.05 to 12.8 μg/mL). The plates were incubated at 37°C for 5 days. All colonies that grew on the drug-containing plates were collected for further analysis.

### Colony instability assay

One SegChrRx1 adaptor (selected at 1.6 μg/mL CSP) was chosen as the representative strain for subsequent experiments due to its prevalence among adapted clones. The adaptor strain was streaked from a –80°C glycerol stock onto a YPD agar plate and incubated at 37°C for 36 hours. A single colony was randomly selected, resuspended in distilled water, and serially diluted. Approximately 200 cells were plated onto a fresh YPD agar plate and incubated again at 37°C for 36 hours. After incubation, one small colony and five large colonies were randomly picked for further analysis.

### Whole genome sequencing

DNA extraction, library construction and sequencing were performed as described previously (Guo et al., 2024). Briefly, cells were cultured on YPD plates at 30°C (~300 colonies/plate), with only small colonies selected. Colonies were resuspended in 1 mL distilled water, pelleted by centrifugation (1 min, microcentrifuge), and processed for genomic DNA extraction using phenol-chloroform. DNA libraries were prepared by BGI (Wuhan, China) as follows: 1 μg genomic DNA was sheared (Covaris LE220), followed by size selection (300–400 bp), end repair, and adapter ligation using Agencourt AMPure XP-Medium kits. After PCR amplification and purification, single-strand circular DNA libraries were prepared and quality-controlled. Sequencing was performed on the BGISEQ-500 platform using DNA nanoball technology, with 100 bp paired-end reads generated through combinatorial Probe-Anchor Synthesis (cPAS).

Data was visualized using Y_MAP_ (Abbey et al., 2014). Raw fastq files were uploaded to Y_MAP_ (version 1.0) (http://lovelace.cs.umn.edu/Ymap/). Read depth (normalized to that of the diploid parent) is shown on the y-axis on a log2 scale converted to absolute copy numbers (1-4). Copy number is indicated on the vertical axis such that trisomy is reflected in black bars above the lengthwise 2N midpoint of each chromosome. Homologs A and B for each chromosome based on the reference genotype are indicated by cyan and magenta, respectively. Heterozygous positions are marked in grey. Allelic ratios (A:B) are color-coded: grey, 1:1 (A/B); cyan, 1:0 (A or A/A); magenta, 0:1 (B or B/B); purple, 1:2 (A/B/B); blue, 2:1 (A/A/B); light blue, 3:1 (A/A/A/B)). Read depth was plotted as a function of chromosome position using the Assembly 22 version of the SC5314 reference genome (http://www.candidagenome.org/download/sequence/*C_albicans*_SC5314/Assembly22/current/*C_albicans*_SC5314_A22_current_chromosomes.fasta.gz).

### RNA-seq

Strains were streaked onto YPD plates from the -80˚C freezer. After 36h incubation at 37°C, several colonies of similar sizes were chosen. Colonies were suspended in distilled water and adjusted to 1x10^4^ cells/mL. 100 µL of cell suspension were spread on YPD plates. The plates were incubated at 37°C for 36 hours. Cells were collected by centrifugation, washed and flash frozen in liquid nitrogen. Total RNA extraction and purification, library construction, and sequencing were performed as described in (Yang et al., 2013). Briefly, total RNA was extracted and purified using the RNeasy Mini Kit (Qiagen, Hilden, Germany). RNA concentration was quantified using a Qubit 4.0 fluorometer (Thermo Fisher Scientific, Waltham, USA), and integrity was assessed with the Agilent 4200 system (Agilent Technologies, Waldbronn, Germany). Libraries were prepared using the ALFA-SEQ RNA Library Prep Kit (Findrop Biosafety Technology, Guangzhou, China), and sequencing was performed on an Illumina MiSeq i100 platform (Illumina,San Diego, USA).

Raw sequence files (.fastq files) underwent quality control analysis using the FastQC tool (http://www.bioinformatics.babraham.ac.uk/projects/fastqc). Reads were mapped to the *C. albicans* SC5314 reference genome (http://www.candidagenome.org/download/sequence/*C_albicans*_SC5314/Assembly22/current/). Differential gene expression profiling was carried out using DESeq2 (Love et al., 2014) with standard parameters. Genes with relative fold changes >1.5 and false discovery rate (FDR)-adjusted P-values <0.05 were considered differentially expressed.

### Data availability

The sequencing data have been deposited in the ArrayExpress database at EMBL-EBI (www.ebi.ac.uk/arrayexpress) under accession number E-MTAB-12336 (DNA-Seq) and E-MTAB-12323 (RNA-Seq).

## Results

### Paradoxical growth of *C. albicans* in the presence of echinocandins

To assess PG in *C. albicans*, we performed serial dilution spot assays of reference strain SC5314 on YPD agar plates containing graded concentrations of three echinocandins: CSP, ANF, and MCF. Following 48-hour incubation at 37°C, SC5314 consistently exhibited PG with all three compounds. Complete growth inhibition occurred at 0.1-0.2 μg/mL CSP and 0.025-0.05 μg/mL ANF/MCF, while growth restoration was observed at 0.4-12.8 μg/mL CSP, 0.1 and 12.8 μg/mL ANF and MCF, respectively. Notably, a subset of colonies developed papillary morphologies (**Figure 1A**), suggesting potential selection of resistant subpopulations through mutational adaptation.

The broth microdilution assay revealed MIC values of 0.025 μg/mL for ANF, 0.05 μg/mL for CSP, and 0.025 μg/mL for MCF. No fungal growth was observed at 0.025–0.1 μg/mL (ANF), 0.05–0.4 μg/mL (CSP), or 0.025–0.1 μg/mL (MCF). However, PG occurred at higher concentrations, specifically within 0.2–12.8 μg/mL (ANF), 0.8–12.8 μg/mL (CSP), and 0.2–12.8 μg/mL (MCF) (**Figure 1B**).

### Roles of PKC, calcineurin, TOR, and Hsp90 in CSP-induced paradoxical growth in *C. albicans*

We investigated the roles of the PKC, calcineurin, and TOR pathways, as well as Hsp90, in PG. The canonical PKC cell wall integrity pathway in yeast consists of a phosphorylation cascade involving *PKC1* (upstream kinase), *BCK1* (MAPKKK), *MKK2* (MAPKK), and *MKC1* (terminal MAPK), which activates transcription factors *RLM1* and the *SWI4*/*SWI6* (SBF) complex to regulate stress response genes, cell wall biosynthesis, and cell cycle progression (LaFayette et al., 2010). The calcineurin signaling pathway comprises the heterodimeric calcineurin phosphatase (encoded by *CMP1*/catalytic subunit and *CNB1*/regulatory subunit) and its downstream transcription factor *CRZ1* (Park et al., 2019). The Hsp90 and TOR pathways can be selectively inhibited using well-characterized pharmacological agents: NVP-HSP990 and geldanamycin (Hsp90 inhibitors), and rapamycin and AZD8055 (TOR pathway inhibitors) (Raught et al., 2001; Menezes et al., 2012).

Here we found, supplementation with the Hsp90 inhibitors NVP-HSP990 (1 μg/mL) and geldanamycin (0.5 μg/mL), or the TOR inhibitor rapamycin (10 ng/mL) and AZD8055 (4 μg/mL), increased *C. albicans* SC5314 susceptibility to CSP (growth inhibition at 0.025 and 0.5 μg/mL CSP, respectively) and abolished PG (**Figure 2A**). Control experiments confirmed that the inhibitor concentrations used (1 μg/mL NVP-HSP990, 0.5 μg/mL geldanamycin,10 ng/mL rapamycin, 4 μg/mL AZD8055) did not affect growth when administered alone, with no inhibition observed even at 10× higher concentrations (data not shown). Similarly, deletions of PKC pathway genes (*MKK2*, *MKC1*, *SWI4*) increased CSP susceptibility and abolished PG, whereas *SWI6* deletion abolished PG without altering susceptibility. In contrast, *RLM1* deletion neither altered CSP susceptibility nor abolished PG. Deletion of calcineurin components *CMP1* or *CNB1* completely abolished PG and increased susceptibility (growth inhibition at 0.05 μg/mL CSP). While *CRZ1* deletion similarly increased susceptibility, it only attenuated - rather than eliminated - PG, with residual growth observed at the highest CSP concentration (12.8 μg/mL). This suggests calcineurin regulates PG primarily through *CRZ1* but retains some *CRZ1*-independent capacity to mediate the response (**Figure 2A**).

In broth microdilution assay, the wild type strain SC5314 exhibited PG in the presence of CSP, while strains with deletions of PKC pathway genes (*MKK2*, *MKC1*, *SWI4*, *SWI6*) and calcineurin pathway genes (*CMP1*, *CNB1*, *CRZ1*) did not exhibit PG. But *rlm1 Δ/Δ* strain still had PG (**Figure 2B** left panel).

In checkerboard assay, SC5314 was grown in YPD broth supplemented with or without NVP-HSP990 (Hsp90 inhibitor) and rapamycin (Tor pathway inhibitor). NVP-HSP990 alone did not exhibit inhibitory effect against SC5314 at the concentrations of 0.25-16 μg/mL. Addition of 0.5-16 μg/mLNVP-HSP990 abolished PG, but addition of 0.25 μg/mL failed to abolish PG (**Figure 2B** middle panel). Rapamycin alone at 40–160 ng/mL had inhibitory effect against SC5314. Addition of subinhibitory concentrations of rapamycin (2.5–20 ng/mL) abolished PG (**Figure 2B** right panel).

Collectively, our findings demonstrate that Hsp90, TOR, PKC, and calcineurin are all essential for maintaining PG in *C. albicans*.

The discontinuous growth patterns in *MKK2* and *MKC1* deletion strains reflect stochastic emergence of drug-tolerant variants. While the papillary colony morphology strongly suggests mutational resistance, comprehensive genomic analysis would be required to definitively exclude other adaptation mechanisms.

### Paradoxical growth is linked to aneuploidy formation

PG in *C. albicans* may result from stress response mechanisms, including compensatory increases in cell wall chitin content (Walker et al., 2013) and the formation of aneuploid subpopulations (Li et al., 2022). To investigate whether exposure to CSP induces genetic adaptations that contribute to PG, we plated approximately 50 cells of the reference strain SC5314 on YPD agar containing CSP concentrations ranging from 0.05 to 12.8 μg/mL (**Figure 3A**). No colonies appeared on plates containing 0.1–0.2 μg/mL CSP, which we define as “lower supra-MIC concentrations”. A few colonies were observed on plates containing 0.4–12.8 μg/mL CSP, which we refer to as “higher supra-MIC concentrations”. From these plates, we isolated all 163 colonies that emerged at CSP concentrations between 0.4-12.8 μg/mL. Subsequent spot assays using 0.1 μg/mL CSP identified 29 colonies (termed “adaptors”) that demonstrated enhanced growth compared to the parental SC5314 strain (data not shown). Whole-genome sequencing analysis of these adaptors revealed they could be categorized into five distinct classes based on their karyotypic alterations. The majority of adaptors (Class 1, n=20) exhibited segmental monosomy of Chromosome R (SegChrRx1), with the deleted region spanning from the ribosomal DNA (rDNA) repeats on the right arm to the right telomere. These Class 1 adaptors were recovered across a broad range of CSP concentrations (1.6, 3.2, 6.4 and 12.8 μg/mL). Class 2 adaptors (n=4) displayed the same SegChrRx1 alteration plus trisomy of Chromosome 2 resulting from amplification of the B homolog (Chr2x3, ABB) and were isolated at 3.2 and 12.8 μg/mL CSP. Class 3 adaptors (n=2) showed SegChrRx1 with an alternative Chr2 trisomy pattern (AAB) and were selected at 3.2 and 6.4 μg/mL CSP. Class 4 adaptors (n=2) had SegChrRx1 combined with trisomy of Chromosome 3 (Chr3x3, AAB) and were recovered at 3.2 μg/mL CSP. The single Class 5 adaptor contained SegChrRx1 along with trisomy of the remaining portion of Chromosome R (SegChrRx3) and was isolated at 6.4 μg/mL CSP. Collectively, all adaptor strains shared SegChrRx1, either as a sole genomic alteration or in combination with additional aneuploidies involving trisomy of Chr2, Chr3, or the remaining segment of ChrR. (**Figure 3B**). Representative spot assays from each adaptor class demonstrated their ability to grow across the full range of CSP concentrations tested (0.1-12.8 μg/mL), in contrast to the parental SC5314 strain which exhibited characteristic PG - showing growth inhibition at 0.1 and 0.2 μg/mL CSP but resumed growth at higher concentrations (0.4-12.8 μg/mL) (**Figure 3C**). Broth microdilution assays showed that the parental strain SC5314 had clear wells at 0.1–0.4 μg/mL CSP, indicating an MIC of 0.1 μg/mL. In contrast, all five adaptor classes exhibited turbid growth across the entire range tested (0.1–12.8 μg/mL CSP), suggesting an MIC of ≥12.8 μg/mL (**Figure 3D**). These results demonstrate that the adaptors are resistant to caspofungin.

Clinical resistance to caspofungin in *C. albicans* is primarily mediated by point mutations in the conserved hotspot regions of *FKS* genes (Perlin, 2015). To determine whether similar mutations arose in our adaptor strains, we examined all three *FKS* genes (*GSC1*, *GSL1*, and *GSL2*) using the Integrative Genomics Viewer (IGV). After visually inspecting the sequencing reads across all 29 adaptor strains, we found no evidence of mutations in any of the FKS genes.

### Unstable resistance to CSP is due to reversible copy number variation

Aneuploidy represents an unstable genetic state that often reverts spontaneously to euploidy in the absence of selective pressure (Yang et al., 2023). To evaluate this genomic instability, we analyzed a SegChrRx1 adaptor strain that exhibited bimodal colony morphology on YPD plates, with distinct small (1.99 ± 0.14 mm diameter) and large (2.59 ± 0.27 mm diameter) colony populations (**Figure 4A**). This size variation likely reflects differential adaptation states, where smaller colonies may maintain the aneuploid genotype while larger colonies represent potential revertants. Whole-genome sequencing of randomly selected colonies (one small and five large) revealed that the small colony maintained SegChrRx1 and retained the ability to grow at lower supra-MIC concentrations of CSP (0.1 and 0.2 μg/Ml), while all five large colonies had reverted to euploidy and lost the ability of growth in the presence of lower supra-MIC CSP concentrations. Both small and large colonies could grow at higher supra-MIC CSP concentrations (**Figures 4B–D**). These findings demonstrate that CSP resistance is mediated by reversible copy number variation of the monosomic region on Chromosome R.

### SegChrRx1 regulates multiple genes associated with CSP resistance

As described above, the parental strain SC5314 exhibits classic PG, showing inhibition at intermediate CSP concentrations (0.1-0.2 μg/ml) but resuming growth at higher concentrations. In contrast, the aneuploid adaptor strains grow across all tested CSP concentrations (**Figure 3**), including the 0.1-0.2 μg/mL range that inhibits the parent. Importantly, when these adaptors revert to euploidy, they regain the parental PG phenotype (**Figure 4**), demonstrating that their extended growth range is directly linked to their aneuploid state. To identify the molecular basis for this phenotypic difference, we performed comparative transcriptomic analysis between a representative SegChrRx1 adaptor strain and the parental SC5314. This approach allowed us to distinguish gene expression changes associated with the aneuploidy-mediated growth capacity from those involved in the parent strain’s paradoxical growth response.

Our analysis revealed that SegChrRx1 mediates complex transcriptional reprogramming involving multiple CSP response pathways (**Table 1**). Notably, we observed upregulation of *GSC1* (encoding the CSP target protein β-1,3-glucan synthase) and chitin synthase genes (*CHS3*, *CHS4*), consistent with established cell wall remodeling responses to echinocandin exposure. Conversely, the chitinase gene *CHT3* was downregulated, suggesting a coordinated modulation of cell wall homeostasis. Key stress response pathways were also affected, with upregulation of calcineurin components (*CNB1*, *CRZ1*) and PKC signaling elements (*MKC1*, *SWI4*). Within the monosomic ChrR region, we identified five dosage-sensitive genes (*ALO1*, *MAL2*, *AHA1*, *SPT3*, *PRE8*) that were downregulated and, according to the Candida Genome Database (Lew-Smith et al., 2025), are associated with CSP resistance when deleted. This observation suggests that reduced gene dosage in this region may contribute to the adapted phenotype through haploinsufficiency effects.

**Table 1:** Differentially expressed genes in SegChrRx1 as compared to parent.

Systematic name	Gene	Chromosome	Ratio (allele A; allele B)
			SegChrRx1/Parent
β-1,3-glucan synthase genes			
*C1_02420C*	*GSC1*	Chr1	3.55; 3.54*
*C1_05600W*	*GSL1*	Chr1	1.03; 0.96
*CR_00850C*	*GSL2*	ChrR	0.83; 0.83
Chitin synthase genes			
*C7_02770W*	*CHS1*	Chr7	0.92; 0.82
*CR_09020C*	*CHS2*	ChrR	1.39; 1.39
*C1_13110C*	*CHS3*	Chr1	2.00; 2.16*
*C3_05700W*	*CHS4*	Chr3	1.27;1.25*
*C2_04140W*	*CHS5*	Chr2	0.96; 0.91
*C7_03060C*	*CHS6*	Chr7	1.02; 1.28
*C1_06010W*	*CHS7*	Chr1	1.32; 1.24
*C3_00710W*	*CHS8*	Chr3	1.20; 0.97
Chitinase genes			
*CR_00180C*	*CHT1*	ChrR	1.04; 1.04
*C5_04130C*	*CHT2*	Chr5	0.93; 0.86
*CR_10110W*	*CHT3*	ChrR	0.58; 0.58*
*C2_02010C*	*CHT4*	Chr2	0.81; 1.36
Calcineurin genes			
*C1_00730C*	*CMP1*	Chr1	1.19; 1.52
*C5_05160C*	*CNB1*	Chr5	1.49*; 1.19
*C3_05780C*	*CRZ1*	Chr3	1.33; 1.33*
PKC genes			
*C3_04470W*	*PKC1*	Chr3	1.09; 1.09
*C7_02990W*	*BCK1*	Chr7	1.19; 1.31
*C2_05780C*	*MKK2*	Chr2	1.18; 1.11
*CR_00120C*	*MKC1*	ChrR	1.36; 1.36*
*C1_01790W*	*SWI4*	Chr1	1.45; 1.50*
*C1_08670W*	*SWI6*	Chr1	0.97; 1.20
*C4_01260W*	*RLM1*	Chr4	1.39; 1.09
ChrR genes in the segmental monosomy region			
*CR_09790W*	*ALO1*	ChrR	0.56; 0.56*
*CR_10790W*	*MAL2*	ChrR	0.19; 0.19*
*CR_10270C*	*AHA1*	ChrR	0.21; 0.21*
*CR_10450C*	*SPT3*	ChrR	0.42; 0.42*
*CR_09380W*	*PRE8*	ChrR	0.53; 0.53*

^*^q<0.05. Bold values are siginificantly differential genes.

### Divergent mutation frequencies at supra-MIC versus paradoxical growth concentrations of caspofungin in *C. albicans*

**Figure 5:**
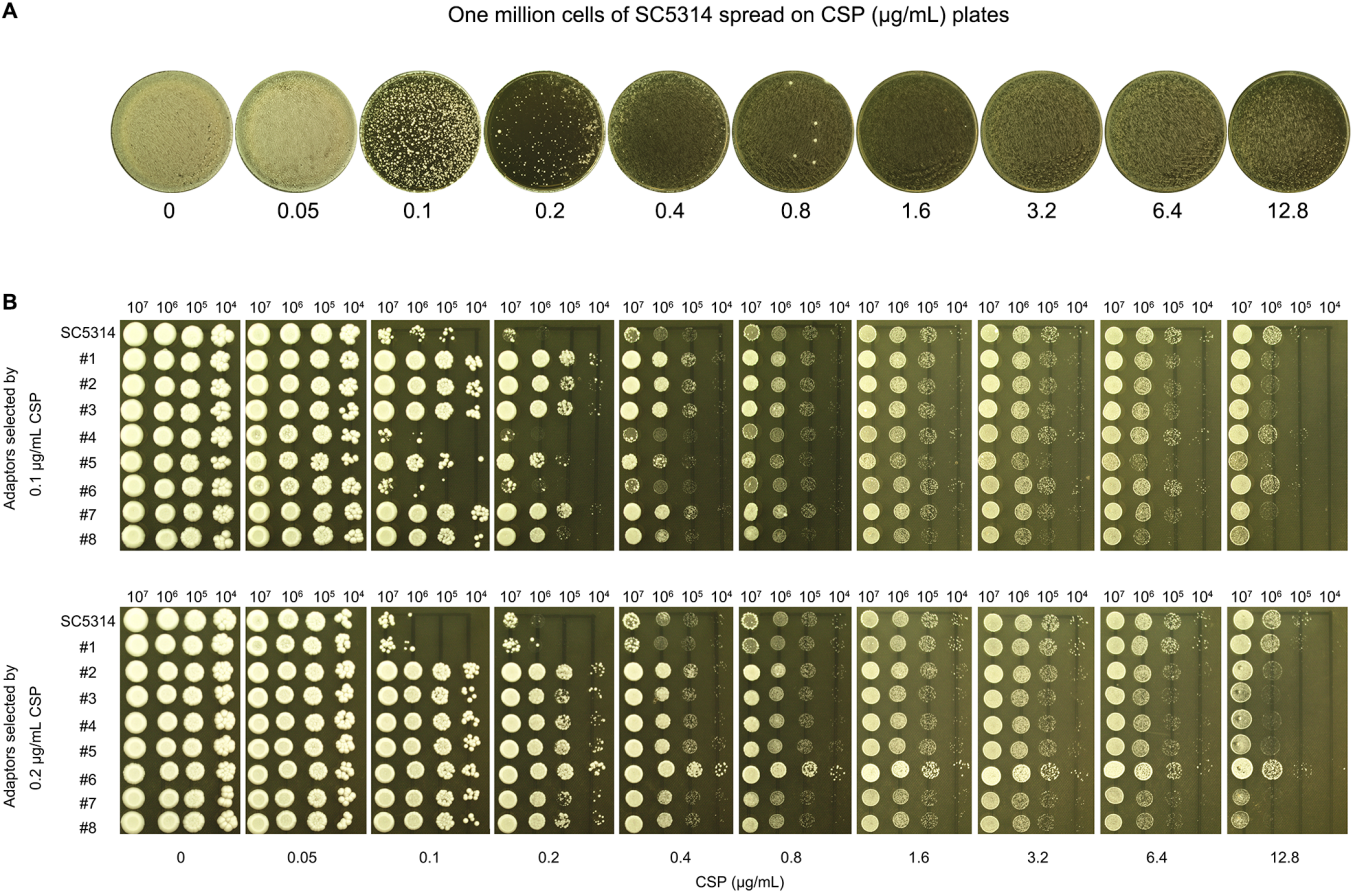
High-frequency selection of CSP-tolerant mutants at supra-MIC concentrations **(A)** Colony formation assay: One million cells of strain SC5314 spread on YPD agar plates containing CSP concentrations (0.05-12.8 μg/mL). The plates were incubated at 37°C for 72h. **(B)** Spot assays: Eight colonies each from 0.1 μg/mL and 0.2 μg/mL CSP plates **(A)** were spotted in serial dilution onto YPD agar with CSP (0.05-12.8 μg/mL). The plates were incubated at 37°C for 72h.

As previously described, when fewer than one hundred cells of the SC5314 were spread on CSP plates, colonies formed exclusively on plates containing 0.4-12.8 μg/mL CSP, with no colonies observed at 0.1 or 0.2 μg/mL CSP. Among the 163 colonies that emerged across these plates, only 29 colonies (17.8%) selected by 1.6-12.8 μg/mL CSP exhibited CSP resistance. In contrast, when one million SC5314 cells were plated, the 0.1 and 0.2 μg/mL CSP plates each produced several hundred colonies, while plates containing 0.4-12.8 μg/mL CSP showed lawn growth (**Figure 5A**). From the colonies appearing on 0.1 and 0.2 μg/mL CSP plates, we randomly selected and tested 16 colonies (8 from each concentration). We found that 6 out of 8 colonies (75%) from the 0.1 μg/mL plates and 7 out of 8 colonies (87.5%) from the 0.2 μg/mL plates demonstrated CSP resistance, confirmed by their ability to grow in the presence of 0.1 and 0.2 μg/mL CSP respectively. Importantly, all 16 tested colonies maintained PG phenotypes (**Figure 5B**). These results demonstrate that the frequency of induced CSP-tolerant mutations is substantially higher at 0.1-0.2 μg/mL compared to concentrations of 0.4 μg/mL and above.

### PG-deficient isolate adapts only to lower supra-MIC concentrations of CSP

A PG-deficient clinical isolate, G309, was tested for adaptation to CSP using two approaches.

**Figure 6:**
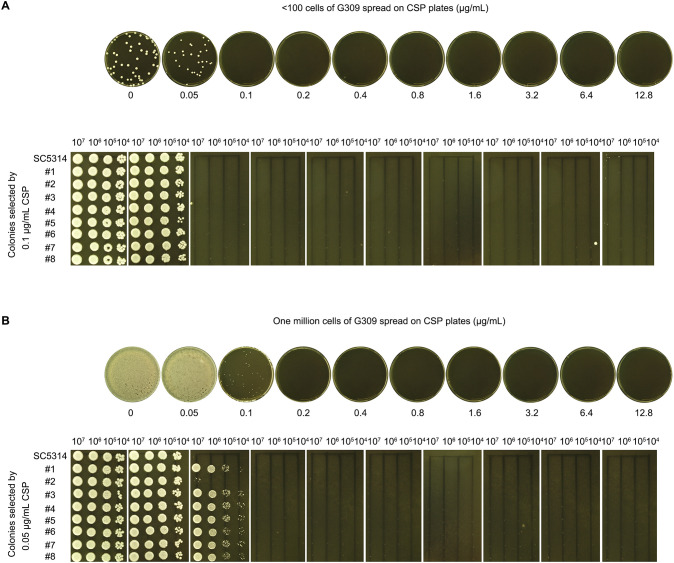
Adaptation of PG-deficient isolate to CSP **(A)** Low-density plating: <100 G309 cells spread on YPD agar plates containing CSP (concentrations are shown in the figure). Colonies from 0.05 μg/mL plate isolated for spot assay. **(B)** High-density plating: 10^6^ G309 cells spread on CSP plates. Colonies from 0.1 μg/mL plate isolated for spot assay. For both panels, all plates incubated at 37°C for 72 h before imaging.

In Approach #1, fewer than one hundred G309 cells were spread on CSP plates (0.05–12.8 μg/mL). Colonies appeared only on the control (no drug) and 0.05 μg/mL CSP plates (**Figure 6A**, top panel). Eight randomly selected colonies from the drug plates were tested using a spot assay, but none exhibited growth on 0.05–12.8 μg/mL CSP plates (**Figure 6A**, bottom panel).

In Approach #2, one million G309 cells were spread on CSP plates. Lawn growth occurred on both the control and 0.5 μg/mL CSP plates. A few colonies appeared on the 0.1 μg/mL CSP plate, but no colonies appeared on the 0.2–12.8 μg/mL CSP plates (**Figure 6B**, top panel). Eight randomly selected colonies from the 0.1 μg/mL CSP plate were tested with a spot assay. All colonies grew on the 0.1 μg/mL CSP plate but failed to grow on 0.2–12.8 μg/mL CSP plates (**Figure 6B**, bottom panel).

## Discussion

Our study demonstrates that CSP paradoxical growth in *C. albicans* depends on stress pathway activation and facilitates transient echinocandin resistance through adaptive aneuploidy. While PG has long been recognized as a stress-responsive phenomenon, our work provides the first direct evidence that segmental aneuploidy—specifically, SegChrRx1—plays a pivotal role in mediating reversible resistance to CSP. This finding represents a significant advance in understanding the genetic and cellular processes that underpin fungal drug resistance.

Our findings demonstrate that Hsp90, TOR, PKC, and calcineurin pathways all play essential but mechanistically distinct roles in CSP PG in *C. albicans*. Pharmacological inhibition of Hsp90 (NVP-HSP990) or TOR (rapamycin) abolished PG while increasing basal CSP susceptibility, suggesting these chaperone and nutrient-sensing pathways maintain both general stress resistance and PG-specific responses. Genetic dissection revealed hierarchical organization within the PKC pathway, where upstream MAPK components (*MKK2*/MKC1) and *SWI4* governed both susceptibility and PG, while *SWI6* deletion only affected PG - indicating functional divergence within the SBF transcription complex. In contrast, calcineurin pathway deletions showed identical phenotypes with complete PG loss and intermediate susceptibility shifts, suggesting this calcium-dependent pathway operates as a unified module. These differential genetic requirements highlight how PG integrates inputs from multiple stress-responsive systems: Hsp90 likely stabilizes key signaling components, TOR modulates adaptive responses, PKC coordinates cell wall remodeling through both MAPK and SBF-dependent outputs, while calcineurin provides a central regulatory hub, together enabling *C. albicans* to overcome CSP stress at supra-inhibitory concentrations.

Strikingly, our genomic analyses reveal that segmental aneuploidy, particularly SegChrRx1, is a recurrent and dominant feature among CSP-resistant isolates. This structural alteration confers broad-spectrum growth across inhibitory CSP concentrations and defines a previously unrecognized route to PG. The identification of five distinct karyotypic classes, all sharing SegChrRx1, suggests that this chromosomal change serves as a core adaptive strategy that may facilitate subsequent acquisition of additional aneuploidies to enhance fitness. Notably, SegChrRx1 is not a fixed genetic trait but a reversible, dynamic adaptation—supporting the concept of aneuploidy as a transient survival mechanism under antifungal pressure (Yang et al., 2023).

Transcriptomic profiling revealed extensive transcriptional rewiring in SegChrRx1-bearing strains, encompassing both direct and indirect effects. On one hand, increased expression of drug target genes (e.g., *GSC1*), chitin biosynthesis enzymes, and components of the calcineurin and PKC pathways collectively support enhanced stress resistance. On the other hand, downregulation of five dosage-sensitive genes within the deleted ChrR region—*ALO1*, *MAL2*, *AHA1*, *SPT3*, and *PRE8*—likely contributes to resistance through haploinsufficiency. These genes are annotated as CSP resistance modulators in *C. albicans* (Lew-Smith et al., 2025), and their decreased dosage appears to recapitulate drug-adapted phenotypes. These findings underscore the nuanced regulatory potential of segmental aneuploidy, which enables coordinated tuning of stress networks and core cellular processes.

The reversibility of CSP resistance following the loss of SegChrRx1 further implicates aneuploidy as a key driver of non-heritable, yet stable, drug adaptation. This phenomenon blurs the line between resistance and tolerance, emphasizing the need to consider chromosomal instability in antifungal susceptibility testing and resistance monitoring. While resistance mutations in *FKS* genes are well-established (Perlin, 2015), our results highlight an alternative and potentially more plastic mechanism of survival—one that evades current diagnostic paradigms focused solely on point mutations.

Clinically, the implications are significant. Aneuploidy-mediated PG may underlie persistent or relapsing infections despite echinocandin therapy, particularly in immune-compromised hosts where fungal stress responses are hyper-activated. Targeting the stress response hubs (e.g., Hsp90 or calcineurin) or pathways that stabilize aneuploidy may represent novel therapeutic strategies. Importantly, combination regimens involving echinocandins and Hsp90 or TOR inhibitors may suppress PG and improve treatment outcomes, as suggested by our inhibitor assays and recent synergistic drug studies (Cowen et al., 2009; Lefranc et al., 2024).

## Conclusions

In conclusion, our work establishes that caspofungin paradoxical growth in *C. albicans* hinges on stress pathway activation for immediate survival, while transient segmental aneuploidy—particularly ChrR monosomy—mediates unstable echinocandin resistance. These findings redefine paradoxical growth as a gateway to adaptive, yet reversible, resistance mechanisms. Future studies should investigate the clinical prevalence of stress-induced aneuploidy and its role in echinocandin treatment failure, offering insights for antifungal strategies targeting genomic instability or stress response pathways.

## Data Availability

The datasets presented in this study can be found in online repositories. The names of the repository/repositories and accession number(s) can be found in the article/**Supplementary Material**.
