# Supplementary figures and images for: Caspofungin paradoxical growth in Candida albicans requires stress pathway activation and promotes unstable echinocandin resistance mediated by aneuploidy
Source: Front Cell Infect Microbiol. 2025 Sep 8;15:1618815. doi: 10.3389/fcimb.2025.1618815 (PMC12450983; doi:10.3389/fcimb.2025.1618815)

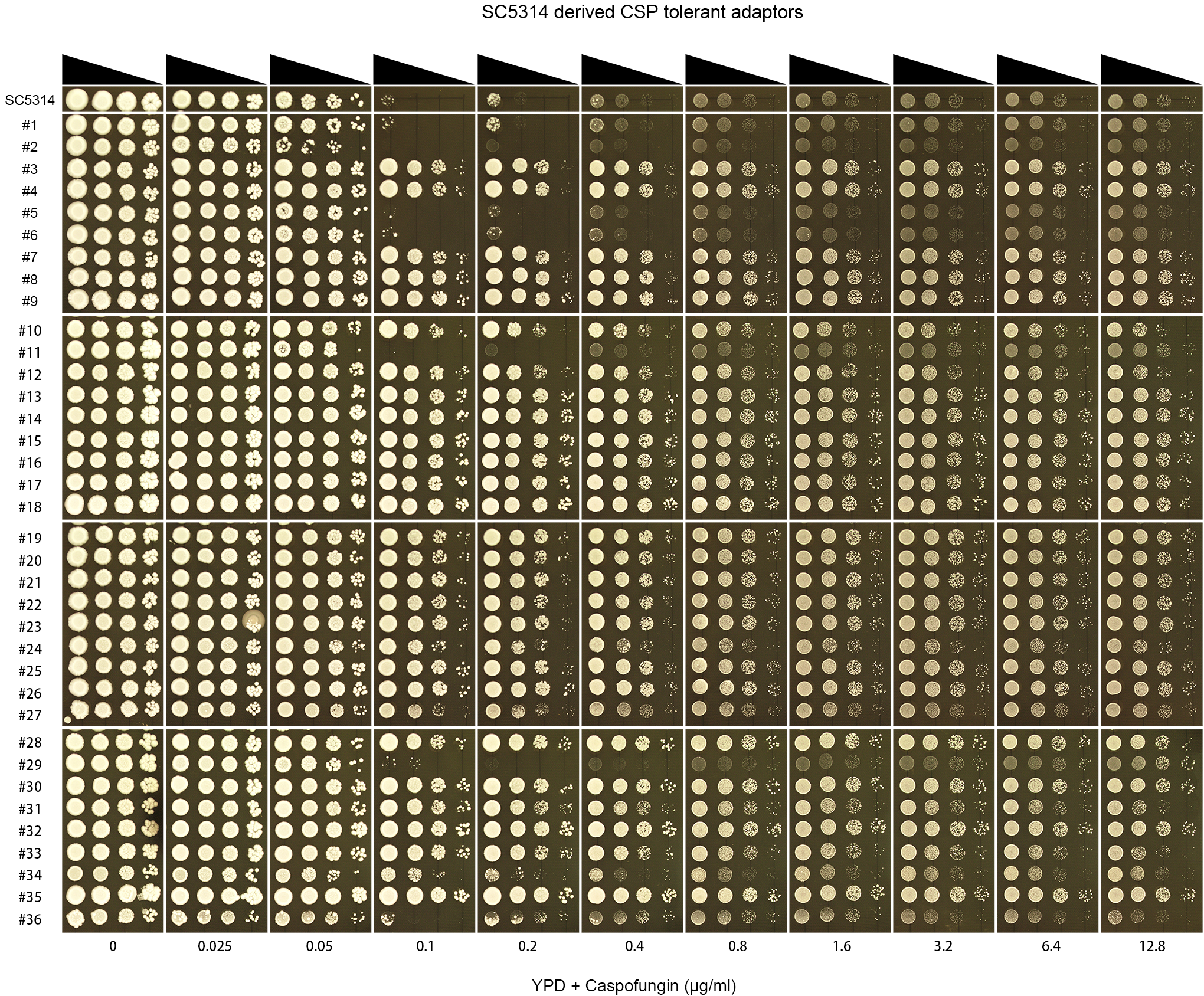

Supplement: Supplementary file 1 [file Image1.tif]
