# Supplementary material for: Caspofungin paradoxical growth in Candida albicans requires stress pathway activation and promotes unstable echinocandin resistance mediated by aneuploidy
Source: Front Cell Infect Microbiol. 2025 Sep 8;15:1618815. doi: 10.3389/fcimb.2025.1618815 (PMC12450983; doi:10.3389/fcimb.2025.1618815)
Supplement: Supplementary file 2 [file Table1.docx]

Table S1. Strains used in this study

| Strain | Parent | Genotype | Source |
| --- | --- | --- | --- |
| SC5314 | Wild type |  | (Yang et al., 2021) |
| YCA892 | SC5314 | mkk2::FRT/mkk2::NAT1 flp | (Yang et al., 2021) |
| YCA1127 | SC5314 | mkc1::FRT/mkc1::NAT1 flp | (Yang et al., 2021) |
| YCA1125 | SC5314 | swi4::FRT/swi4::NAT1 flp | (Yang et al., 2021) |
| YCA1121 | SC5314 | swi6::FRT/swi6::NAT1 flp | (Yang et al., 2021) |
| YCA1126 | SC5314 | rlm1::FRT/rlm1::NAT1 flp | (Yang et al., 2021) |
| YCA641 | SC5314 | cmp1::FRT/cmp1::NAT1 flp | (Yang et al., 2021) |
| YCA623 | SC5314 | cnb1::FRT/cnb::NAT1 flp | (Yang et al., 2021) |
| YCA736 | SC5314 | crz1::FRT/crz1::NAT1 flp | (Yang et al., 2021) |

**Reference**

Yang, F., Gritsenko, V., Slor Futterman, Y., Gao, L., Zhen, C., Lu, H., et al. (2021). Tunicamycin Potentiates Antifungal Drug Tolerance via Aneuploidy in Candida albicans. *mBio* 12(4)**,** e0227221. doi: 10.1128/mBio.02272-21.
